# Enhancing Frozen Storage Stability and Nutritional Quality of Barhi Dates Using Okra Extract and Gelatin‐Based Edible Coatings Combined With Different Packaging Systems

**DOI:** 10.1002/fsn3.72306

**Published:** 2026-08-31

**Authors:** Khalid. N. Asal, Raghad Adnan Ali AL‐Qady, Kamil Atya Abed Sanjari, Kh. A. S. Al‐Hamdani, Zayad Amer Mustafaa, Heidar Meftahizade

**Affiliations:** ^1^ College of Agriculture University of Tikrit Tikrit Iraq; ^2^ Department of Biology, College of Education for Pure Sciences Al‐Hamdaniya University Al‐Hamdaniya Iraq; ^3^ Ministry of Education Kirkuk Directorate of Education Kirkuk Iraq; ^4^ College of Agriculture University of Samarra Samarra Iraq; ^5^ Agricultural Technical College Northern Technical University Mosul Iraq; ^6^ Department of Horticultural Science, Faculty of Agriculture & Natural Resources Ardakan University Ardakan Iran

**Keywords:** date fruits, edible coatings, gelatin, okra extract, postharvest quality

## Abstract

The present study evaluated the effects of edible coatings based on okra extract and gelatin, combined with different packaging systems, on the physicochemical quality and nutritional characteristics of Barhi date fruits during frozen storage at −18°C. A factorial experiment consisting of five coating treatments (uncoated, okra extract 25 and 50 g L^−1^ and gelatin 50 and 100 g L^−1^) and three types of packaging (control, paper bags, polyethylene bags) was conducted in a completely randomized design with three replications. Fruits treated with polyethylene packaging containing 100 g L^−1^ gelatin showed the highest pH (5.83) and the highest total soluble solids (TSS, 45.3%+) compared to the control. The dry matter and carbohydrate contents were highest in fruits treated with 100 g L^−1^ gelatin (53.3% and 15.66%, respectively); this indicated a slower rate of respiration and sugar depletion. The protein content increased from all treatments, reaching 7.92% in fruits treated with 50 g L^−1^ okra extract, while sugar levels were maintained in two fruits treated with either 100 g L^−1^ gelatin (55.42%) or 50 g L^−1^ okra extract with polyethylene packaging (54.7%). Importantly, with the treatment of okra extract at 50 g L^−1^, contents of nitrogen and phosphorus were increased by 42.8% and 14.3%, respectively. Overall, the combined application of edible coatings and polyethylene packaging effectively maintained several quality attributes of Barhi dates during frozen storage.

## Graphical abstract

Okra and gelatin edible coatings preserved Barhi date quality during frozen storage. Gelatin at 100 g L^−1^ with polyethylene packaging showed the best overall performance. Coatings improved dry matter, carbohydrates, protein, and sugar retention. Okra extract increased nitrogen and phosphorus concentrations in stored dates.

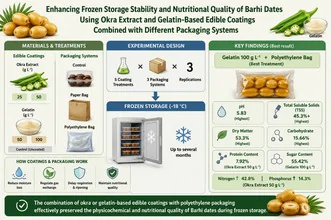

## 1 Introduction

Date palm ( *Phoenix dactylifera* L.), a member of the Arecaceae family, is one of the oldest flowering monocotyledonous plants in the world (Krueger 2021). It has long been regarded as the “Tree of the Arabs” and the “Blessed Tree” for its vital role in the culture, economy, and food security of Middle Eastern countries. Among the numerous cultivars, the Barhi date is highly valued for its golden‐yellow color, sweet flavor, and soft texture, making it one of the most popular and expensive varieties in both local and export markets (Obón et al. 2023).

Despite its nutritional and economic importance, date‐producing regions still face major postharvest challenges, particularly related to short quality preservation during frozen storage and high perishability (Obón et al. 2023). The Khalal stage of the fruit development process is often commercially important because the fruit is a stage with high freshness and sensory quality, but the high moisture content and high respiration rates at this stage can cause softening, weight loss, and other quality losses (Alqahtani et al. 2025). Barhi dates may experience a weight loss of more than 15% if stored in cold conditions over a period of 2 weeks. They may also change in color and become unmarketable. These facts highlight the need to develop natural, safe, and environmentally friendly preservation methods which would prolong the quality preservation during frozen storage of the fresh dates (Selim et al. 2020).

Recently, natural edible coatings have received widespread recognition as a postharvest handling tool to preserve fruit quality (Abd Elwahab et al. 2025; Al‐Habsi 2025; Ali et al. 2025). These coatings, which act as semi‐permeable barriers, limit transpiration, regulate gas exchange, and prolong the ripening process. Okra ( *Abelmoschus esculentus* ) extract is one of the most likely candidates among natural biomass sources. It is a promising candidate because of its mucilage, polysaccharides, and antioxidant compounds which can generate a biopolymeric film that will restrict moisture diffusion and exposure to oxidation (Dantas et al. 2021; Olawuyi and Lee 2022; Zhu et al. 2020).

Additionally, hydrogels identified as the new generation of natural preservation matrices provide an excellent capacity for water retention and create a humid microenvironment around fruits that reduces both weight loss and appearance of fruit (Pillai et al. 2024). The integration of the two systems, okra extract and hydrogel matrix, provides an innovative and synergetic approach to the development of intelligent, bio‐based coatings for the regulation of moisture and gas exchange, modulate respiration, and retain nutritional and sensory qualities of fruits (Saleem et al. 2023). Although edible coatings have been widely investigated under refrigerated storage conditions, little information is available regarding their effectiveness during long‐term frozen storage. Freezing considerably reduces metabolic activity but may also induce cellular damage due to ice crystal formation and moisture migration. Therefore, evaluating edible coatings under frozen conditions is important to determine whether these coatings can minimize quality deterioration during prolonged frozen storage. Thus, the present research aims to improve the quality preservation during frozen storage and commercial value of Barhi dates through okra extract and hydrogel‐based postharvest treatment methods. This is an initiative toward developing sustainable and low‐cost methods to sustain fruit quality and limit food loss in arid and semi‐arid regions.

## 2 Materials and Methods

### 2.1 Sample Collection and Preparation

Barhi date palm fruits ( *Phoenix dactylifera* L.) were collected during the 2021–2022 growing season from a village located in the southwestern region of Kirkuk Governorate, Iraq. Fruits were selected based on uniform size, color, and physiological maturity, while fruits exhibiting any physical damage, cracking, fungal infection, or surface blemishes were discarded. The selected fruits were washed thoroughly with distilled water to remove surface contaminants and air‐dried at room temperature until the moisture on the peel surface had completely evaporated to avoid any delay in treatment application. The plant material (Barhi date palm fruits) was formally identified by Dr. Khalid N. Asal. As Barhi is a widely cultivated commercial date palm cultivar routinely planted by farmers, and not a wild‐collected species, an herbarium voucher specimen is not typically deposited. Fruits were harvested at the Khalal maturity stage based on their characteristic bright yellow color, firm texture, uniform size, and average fruit weight (approximately 15–18 g). Fruits showing any signs of disease, cracking, mechanical injury, or uneven ripening were excluded from the experiment.

### 2.2 Experimental Design

The experiment was conducted as a factorial arrangement in a Completely Randomized Design (CRD) with three replications. Factor A consisted of five coating treatments (uncoated control, okra extract at 25 and 50 g L^−1^, and gelatin at 50 and 100 g L^−1^), whereas Factor B consisted of three packaging treatments (unpackaged control, paper bags, and polyethylene bags). Each treatment combination was randomly assigned to the experimental units. After cleaning, sorting, and applying the respective coating treatments, the fruits were placed into their designated packaging types and stored under freezing conditions at −18°C ± 2°C. Quality parameters were evaluated at the end of the storage period. Each experimental unit consisted of 20 fruits. Three independent replications were used for each treatment combination, resulting in a total of 15 treatment combinations (5 coating treatments × 3 packaging treatments). All treatment combinations were randomly assigned to the experimental units using a completely randomized design. Each replicate was considered an independent experimental unit for statistical analysis.

### 2.3 Preparation and Application of Edible Coatings

Fresh okra pods were washed with distilled water, sliced into small pieces, and mixed with distilled water at the required concentrations (25 and 50 g L^−1^). The mixture was heated at 60°C for 30 min under continuous stirring, cooled to room temperature, filtered through muslin cloth, and the obtained mucilage was used as the coating solution. Gelatin coating solutions were prepared by dissolving food‐grade gelatin in distilled water at concentrations of 50 and 100 g L^−1^ under continuous stirring at 50°C until complete dissolution. Date fruits were individually immersed in each coating solution for 2 min, removed, and allowed to dry at room temperature (25°C ± 2°C) for approximately 2 h until a uniform coating film was formed on the fruit surface before packaging. Polyethylene packaging consisted of low‐density polyethylene (LDPE) bags (20 × 30 cm, thickness 50 μm), whereas paper packaging consisted of commercial kraft paper bags of similar dimensions.

### 2.4 Physicochemical Analyses

#### 2.4.1 Determination of pH

The pH of the date fruit samples was determined according to the AOAC method (Ahmed et al. 2023). Briefly, 10 g of homogenized date flesh was mixed with 100 mL of distilled water and thoroughly homogenized by stirring. The pH of the suspension was measured using a calibrated digital pH meter. The pH meter was calibrated before each series of measurements using standard buffer solutions of pH 4.0 and 7.0. All measurements were performed in triplicate at room temperature (25°C ± 2°C), and the mean values were reported.

#### 2.4.2 Total Soluble Solids (TSS)

Total soluble solids (TSS) were determined using a hand‐held digital refractometer. Before each measurement, the refractometer was calibrated with distilled water at 20°C according to the manufacturer's instructions. Fresh date fruit pulp was homogenized, and a few drops of the extracted juice were placed directly on the prism of the refractometer. The TSS values were recorded as degrees Brix (°Brix), representing the percentage of soluble solids in the juice. After each measurement, the prism was cleaned with distilled water and dried with soft tissue paper. Three independent measurements were performed for each sample, and the results were expressed as the mean ± standard deviation (AOAC 1990).

#### 2.4.3 Moisture and Dry Matter Content

Moisture and dry mass were determined by gravimetric means. The samples were then dried in a forced air oven at 70°C until a constant weight was achieved. Moisture and dry mass percentages were calculated using the equations:

$$\text{Water content}\%=\frac{\text{Fresh Weight}-\mathrm{Dry}\;\text{Weight}}{\text{Fresh Weight}}\times 100$$

$$\mathrm{Dry}\;\text{Matter}\%=\frac{\;\mathrm{Dry}\;\text{Weight}}{\text{Fresh Weight}}\times 100$$

where FW and DW represent the fresh and dry weights, respectively.

#### 2.4.4 Total Carbohydrate Content

Total carbohydrate content was determined using the phenol–sulfuric acid colorimetric method described by (Schanderl 1970). Briefly, 0.2 g of dried fruit powder was hydrolyzed using 1 N hydrochloric acid in a water bath at 60°C for 60 min. The extraction procedure was repeated three times, and the extracts were combined and diluted to 100 mL with distilled water. An aliquot (1 mL) was mixed with 1 mL of 5% phenol solution and 5 mL concentrated sulfuric acid. After color development, absorbance was measured at 490 nm using a UV–Vis spectrophotometer. Glucose was used as the calibration standard, and total carbohydrate content was expressed as percentage of dry weight.

#### 2.4.5 Determination of Crude Protein

Crude protein content was determined using the Kjeldahl method according to the AOAC Official Method 981.10 (AOAC 1990). Briefly, approximately 1 g of dried and ground date fruit sample was digested with concentrated sulfuric acid in the presence of a catalyst until a clear solution was obtained. The digest was neutralized with sodium hydroxide, and the liberated ammonia was distilled and collected in a boric acid solution. The distillate was titrated with standardized hydrochloric acid to determine the nitrogen content. Crude protein was calculated by multiplying the total nitrogen content by a conversion factor of 6.25 and expressed as a percentage of dry weight. All analyses were performed in triplicate.

#### 2.4.6 Determination of Reducing Sugars

Reducing sugars were determined using the 3,5‐dinitrosalicylic acid (DNS) colorimetric method as described by Melton et al. (1970). Briefly, 1 g of homogenized date fruit was extracted with 10 mL of distilled water and centrifuged at 5000 × *g* for 10 min. An aliquot (1 mL) of the supernatant was mixed with 1 mL of DNS reagent and heated in a boiling water bath (100°C) for 5 min to allow color development. The reaction mixture was then cooled to room temperature, and 8 mL of distilled water was added. The absorbance was measured at 540 nm using a UV–Vis spectrophotometer. A standard calibration curve was prepared using glucose solutions of known concentrations, and the reducing sugar content was calculated from the standard curve and expressed as percentage of dry weight. All measurements were performed in triplicate (Melton et al. 1970).

##### 2.4.6.1 Nitrogen

Total nitrogen was determined using the standard Kjeldahl method. Crude protein was calculated as *N* × 6.25 (Goyal et al. 2022).

##### 2.4.6.2 Phosphorus

Phosphorus content was determined spectrophotometrically using the molybdenum blue method following standard procedures for plant tissue analysis (Wieczorek et al. 2022).

### 2.5 Statistical Analysis

The experiment was conducted as a factorial arrangement in a Completely Randomized Design (CRD) with three replications. Factor A consisted of five coating treatments (uncoated control, okra extract at 25 and 50 g L^−1^, and gelatin at 50 and 100 g L^−1^), whereas Factor B consisted of three packaging treatments (unpackaged control, paper bags, and polyethylene bags). Each treatment combination was randomly assigned to the experimental units. All data were subjected to two‐way ANOVA using SAS software (version 9.4). When ANOVA indicated significant effects (*p* ≤ 0.05), mean comparisons were performed using the least significant difference (LSD) test. The LSD test was selected because the experiment involved a balanced factorial design with a limited number of treatment combinations, allowing efficient pairwise comparison following a significant overall ANOVA. Hierarchical clustering and Pearson correlation analyses were conducted using the ClustVis R package (Metsalu and Vilo 2015) to visualize relationships among treatments and quality parameters. Heatmaps and multivariate clustering were generated to support data interpretation. Principal component analysis (PCA) was conducted using XLSTAT 2020 from AddinSoft SARL. The statistical model included the main effects of coating treatment, packaging treatment, and their interaction. The assumptions of normality and homogeneity of variance were verified before conducting ANOVA. *F* values, degrees of freedom (df), and associated probability levels were used to determine treatment significance at *p* ≤ 0.05.

## 3 Results and Discussion

The results of the analysis of variance of the data are given in Table S1.

### 3.1 Effect of Gelatin Coating and Packaging on pH and TSS of Dates

**FIGURE 1:**
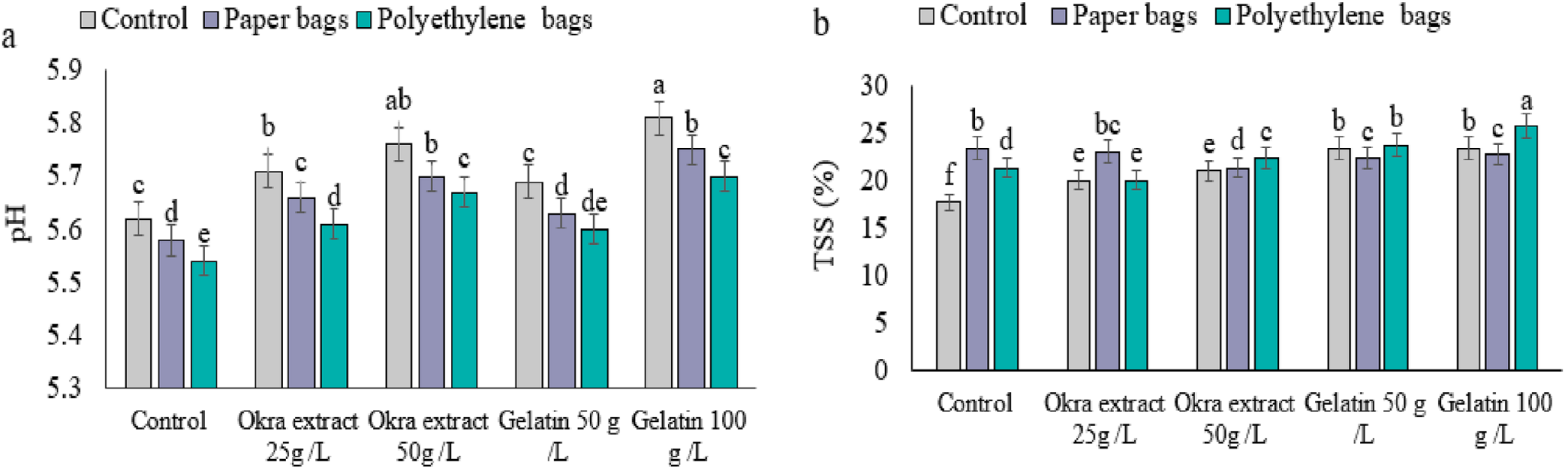
Effect of packaging type (paper bags, polyethylene bags, and uncovered control) and coating treatment (control, okra extract at 25 and 50 g L^−1^, gelatin coating at 50 and 100 g L^−1^) on postharvest quality parameters of Barhi date palm ( *Phoenix dactylifera* L.) fruits: (a) pH, (b) TSS. Data represent mean ± SE (*n* = 3). Means followed by different letters are significantly different at *p* ≤ 0.05 according to the LSD test.

The treatment of 100 g L^−1^ gelatin showed the highest pH (5.83), while the lowest pH (5.54) was observed in fruits treated with 100 g L^−1^ gelatin and packaged in polyethylene bags (Figure 1a), representing a 3.38% decrease compared to the control (Figure 1a). Gelatin and okra coatings act as semi‐permeable barriers, limiting moisture loss, reducing respiration rates, and slowing organic acid degradation (Felicia et al. 2022; Hasdar et al. 2026). Polyethylene packaging can significantly improve fruit quality and increase quality preservation during frozen storage compared to other packaging materials (Mahara and Karki 2024). In our study, the application of edible coatings combined with packaging (such as gelatin + PE) resulted in altered pH dynamics compared to uncoated controls during frozen storage. Comparable results have been observed in refrigerated Barhi date fruits coated with chitosan‐based edible films, where a slight decrease in pH occurred over storage time, indicating that coatings can influence acid stability by limiting metabolic changes linked to acid breakdown and microbial activity. This suggests that edible coatings can play a role in maintaining biochemical stability in dates during postharvest storage. Consistent with our findings, studies on Barhi dates coated with various edible materials including gum arabic also demonstrated that coatings helped preserve chemical properties such as pH and total soluble solids under storage conditions. Specifically, higher concentrations of gum Arabic coating maintained more stable pH values in Barhi dates during cold storage, reinforcing the concept that edible barriers reduce metabolic alterations that influence acidity (Mahara and Karki 2024). The retention of chemical stability in coated dates can also be explained by reduced respiration rates. Organic acids are central substrates in aerobic respiration pathways, and when respiration is slowed due to reduced O_2_ ingress through a coating film, the depletion of internal acids is likewise reduced. This concept has been observed in other fresh produce where edible films decrease respiration and maintain quality parameters, supporting the general mechanistic basis for why pH changes are less pronounced in coated fruits.

The postharvest quality of Barhi date palm fruits is significantly affected by both packaging conditions and edible coating methods, with distinct impacts on physical and chemical attributes during storage. Studies have shown that the choice of packaging material and method can alter moisture loss, textural properties, and biochemical stability of date fruits. Specifically, vacuum packaging combined with high‐barrier films has been demonstrated to reduce weight loss and maintain firmness and moisture content compared to traditional packaging like carton or perforated plastic baskets, by limiting gas and moisture exchange with the external environment and thus reducing respiration and dehydration processes. This mechanism helps preserve fruit quality parameters including pH, total soluble solids (TSS), and water activity (aw), particularly under cold storage conditions (Tsegaye 2020).

Overall, the literature consistently indicates that selection of appropriate packaging materials (high barrier, vacuum or MAP) and optimized edible coating formulations (polysaccharides like gum Arabic or chitosan, alone or combined with functional extracts) can significantly enhance the postharvest quality of Barhi date palm fruits by reducing physical deterioration (weight loss, softening) and stabilizing chemical attributes (pH, TSS, water activity) during storage. These mechanisms are directly relevant to our results and support the interpretation that both packaging and coating strategies exert complementary protective effects on date quality.

TSS content increased from 17.67% in the control to 25.67% in fruits treated with 100 g L^−1^ gelatin with polyethylene bags (Figure 1b). The gelatin film in combination with polyethylene bags retains higher relative humidity and prevents mass loss of the fruit, thereby improving TSS content; this situation benefits fruits with respect to the retention of TSS (Agostini et al. 2013). According to research conducted by (Kumar and Thakur 2020) fruits unpacked in low‐density polyethylene (LDPE) film bags supplemented with sachets of iron powder exhibited the maximum levels of TSS, along with increased levels of fruit quality parameters, at the conclusion of the storage period. This demonstrates that the packaging materials and additives significantly improve the TSS of fruits during storage. According to (Sibomana et al. 2015), the authors also determined that TSS of tomatoes significantly increased as the moisture level decreased. It can also be inferred that packaging type and moisture control are important factors to help sustain and preserve or improve the TSS in fruit that are being stored.

### 3.2 Impact of Coating and Packaging on Fruit Quality

**FIGURE 2:**
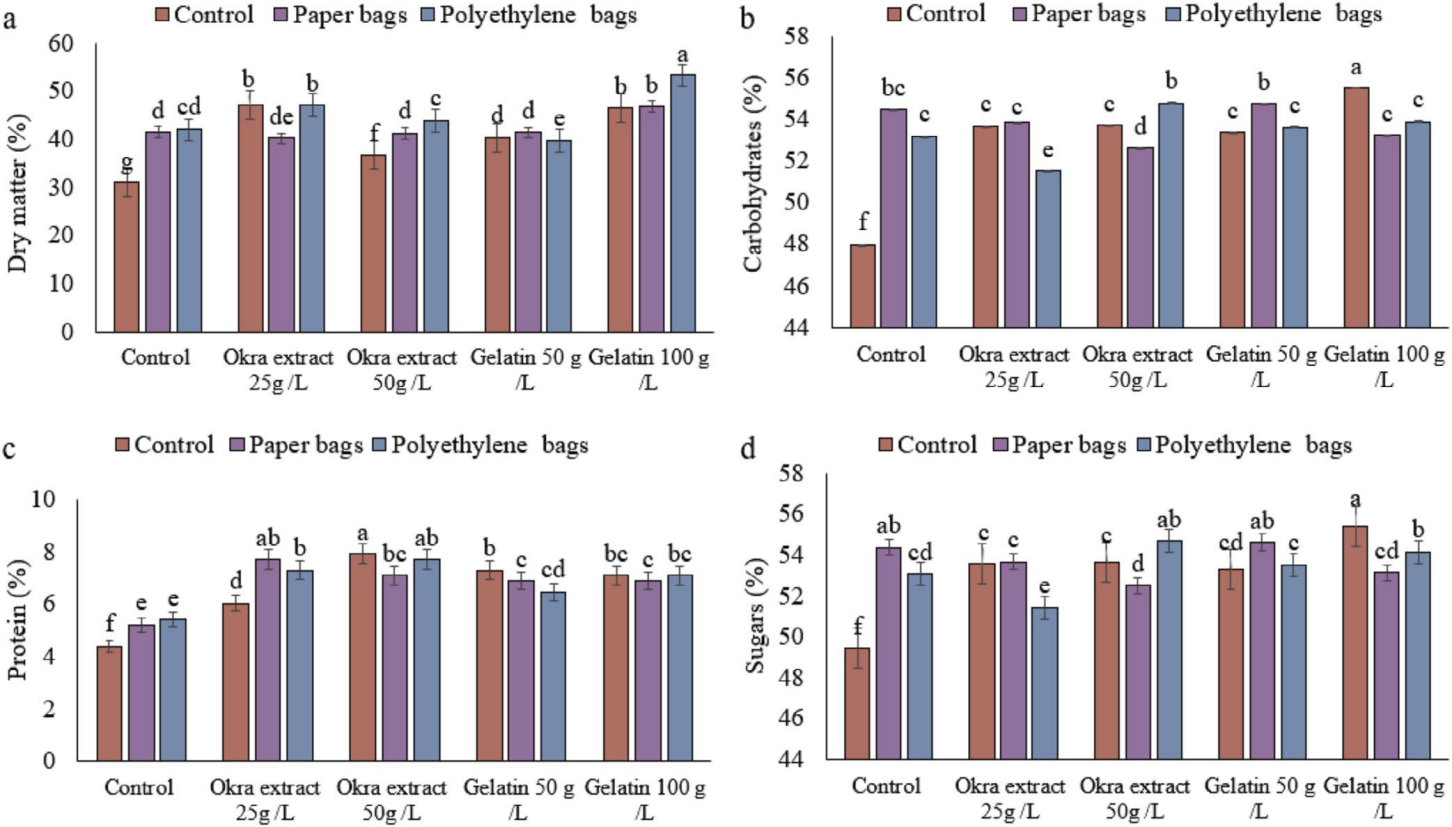
Effect of packaging type (paper bags, polyethylene bags, and uncovered control) and coating treatment (control, okra extract, gelatin coating) on postharvest quality parameters of Barhi date palm ( *Phoenix dactylifera* L.) fruits: (a) Dry matter (b) Carbohydrate (c) Protein and (d) Sugars. Data represent mean ± SE (*n* = 3). Means followed by different letters are significantly different at *p* ≤ 0.05 according to the LSD test.

According to the data, the highest amount of dry matter was in the gelatin treatment with 100 g L^−1^ in a polyethylene bag (53.3%) and the lowest in the control (31.1%). (Figure 2a). Dry matter (DM) is considered a trustworthy indicator of fruit quality and is correlated to consumer preferences for attributes including firmness, crunchiness, juiciness, sweetness, and flavor (Serra et al. 2019). Sorting fruits after the harvest based on their DM content has been shown to assist in predicting internal quality after extended cold storage (Serra et al. 2019). The association between an increased dry matter (DM) content, for example in pears and mangoes corresponds to post‐storage and ripening quality (Santos Neto et al. 2018). The DM content of gelatin stored in polyethylene bags is also influenced by moisture loss over time. The amount of DM content in pineapple fruit was observed to increase with storage time as water escaped from the pulp (Kamol et al. 2014). For example, it was observed at 18 days during the storage period that as a function of storage condition, the control samples had greater dry matter content (13.7%) than those stored in unperforated polyethylene bags (11.7%). This means that polyethylene bags may have helped retain moisture and thereby influence the dry matter content. This study also showed moisture content decreased due to storage time, which is also supported by the increase in dry matter content (Kamol et al. 2014).

Edible coatings, including those made with gelatin, can act as carriers for antioxidants and nutritional fortifiers, potentially enhancing the carbohydrate content of fruits by reducing respiration and moisture loss (El‐Gioushy and Baiea 2020; Zhang, Cui, Wang, Shi, et al. 2021). Gelatin coatings, especially when combined with other substances, have been shown to preserve the nutritional quality of tomatoes and enhance their antimicrobial activity (Frassinetti et al. 2021). In the case of sweet cherries, a carboxymethyl chitosan‐gelatin based edible coating incorporating additives like CaCl_2_ and ascorbic acid significantly delayed fruit decay and maintained quality during storage (Zhang, Cui, Wang, et al. 2021). Furthermore, gelatin treatments on Samany date palm fruits varied in effectiveness, with some treatments significantly reducing fruit weight loss and decay, although the response of other fruit quality parameters like total sugars and acidity to gelatin treatments was less pronounced (El‐Gioushy and Baiea 2020).

### 3.3 Boosting Protein and Sugar Retention in Dates

In this study, the control showed a protein content of 4.4%, while okra extract at concentrations of 25 and 50 g L^−1^ resulted in an increase in protein levels of 6.04% and 7.92% in unpackaged samples, respectively (Figure 2c). Edible coatings formulated with okra extract help reduce moisture loss, respiration rate, and oxidative reactions in fruit tissues (Wang et al. 2024).

The results showed that the highest sugar content was observed in 100 g L^−1^ gelatin (55.42%) compared to the control (49.46%) (Figure 2d). Gelatin coatings have been shown to positively impact the postharvest quality of fruits, such as mangoes, by delaying ripening and maintaining quality attributes like sugar content and total carotenoids (Gol and Rao 2014). In the case of Samany date palm fruits, gelatin treatments at concentrations of 1%, 2%, and 4% were found to produce high fruit total sugar content, comparable to control treatments, indicating their effectiveness in preserving sugar levels during storage (El‐Gioushy and Baiea 2020).

### 3.4 Enhanced Nitrogen and Phosphorus in Dates

**FIGURE 3:**
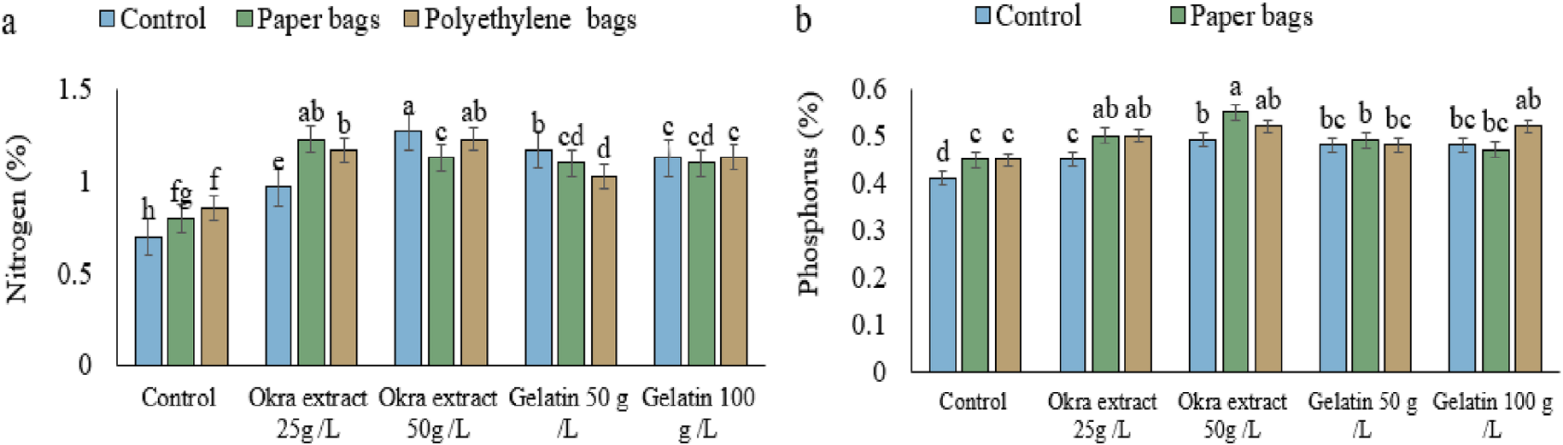
Effect of packaging type (paper bags, polyethylene bags, and uncovered control) and coating treatment (control, okra extract, gelatin coating) on postharvest quality parameters of Barhi date palm ( *Phoenix dactylifera* L.) fruits: (a) Nitrogen and (b) phosphorus. Data represent mean ± SE (*n* = 3). Means followed by different letters are significantly different at *p* ≤ 0.05 according to the LSD test.

Date fruits treated with okra extract had the highest nitrogen content (1.27%) compared to the control (0.7%) (Figure 3a). Similarly, the combination of okra extract and polyethylene packaging resulted in the highest phosphorus content (0.55%), which was 14.34% higher than the control (0.41%) (Figure 3b).

The observed increases in nitrogen and phosphorus concentrations should be interpreted with caution. Because edible coatings reduce water loss during storage, part of the increase may reflect a concentration effect rather than a true accumulation of nutrients. Since nutrient uptake through the fruit epidermis was not investigated in the present study, further physiological studies are required to clarify the underlying mechanism (Uwiringiyimana et al. 2024). Therefore, the present findings should be interpreted as changes in nutrient concentration rather than direct evidence of nutrient absorption by the fruit tissues. Polyethylene packaging helps preserve dates by creating a modified atmosphere that balances O_2_ and CO_2_ transmission, reducing respiration, slowing biochemical reactions, and extending quality preservation during frozen storage (Chonhenchob et al. 2013).

### 3.5 PCA Highlights Key Postharvest Improvements

**FIGURE 4:**
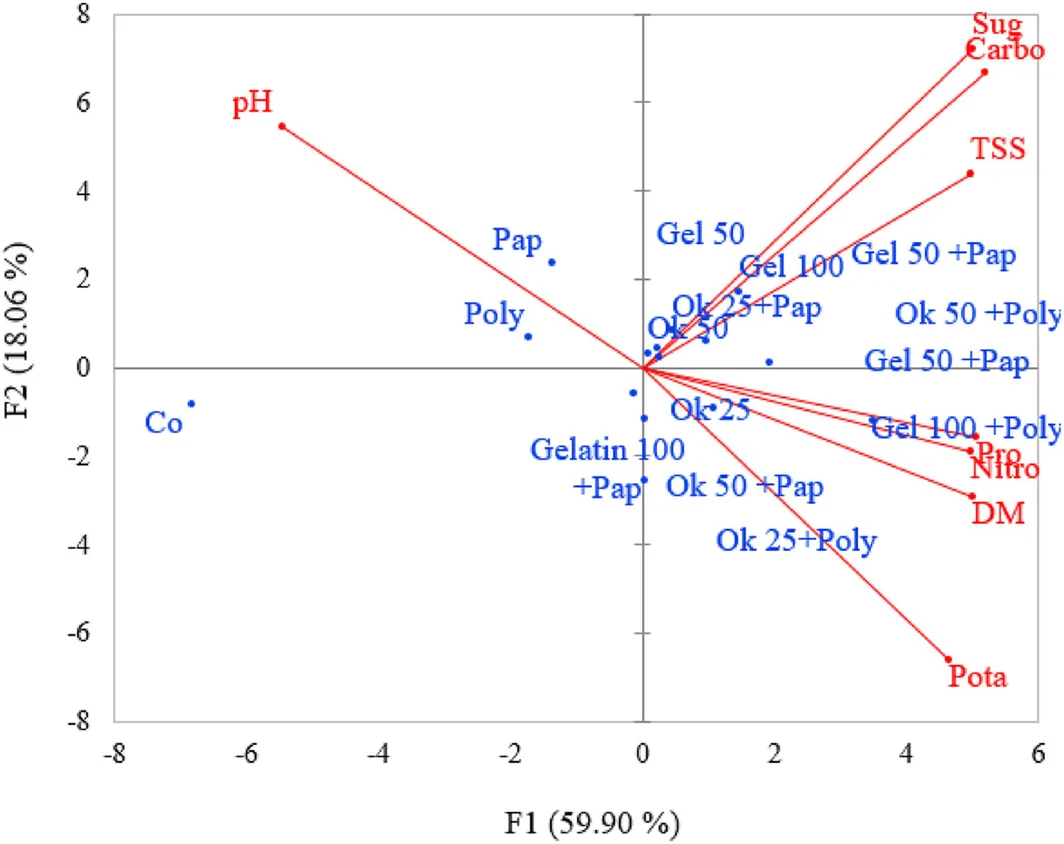
Effect of packaging type (paper bags, polyethylene bags, and uncovered control) and coating treatment (control, okra extract, gelatin coating) on postharvest quality parameters of Barhi date palm ( *Phoenix dactylifera* L.) fruits: Principal component analysis (PCA). Parameters include: PH; TSS; Sug: Sugar; Carbo; carbohydrate; Pro: Protein; DM: Dry matter; Nitro: Nitrogen; Pho: Phosphorus, Gel: Gelatin; Poly: Polyethylene bags; Pap: Paper bags; Ok: Okra.

Applications of principal component analysis (PCA) revealed identifiable relationships between coating and packaging treatments and the quality attributes of the fruit. Certain treatment combinations, including gelatin 50 g L^−1^, gelatin 100 g L^−1^, Okra 25 g L^−1^ with paper bags, Okra 50 g L^−1^, gelatin 50 g L^−1^ with paper bags, Okra 50 g L^−1^ with polyethylene bags, and polyethylene bags with gelatin 50 g L^−1^, were positively associated with sugar, carbohydrate, and TSS in the fruit, indicating enhanced retention of sweetness and carbohydrates (Figure 4).

Other treatment combinations, such as polyethylene bags with gelatin 100 g/L, Okra 25 g L^−1^, paper bags with Okra 50 g L^−1^, gelatin 100 g L^−1^ with paper bags, and Okra 25 g L^−1^ with polyethylene bags, showed positive associations with overall postharvest quality. Additionally, packaging alone (polyethylene and paper bags) was associated with higher pH values, suggesting an influence on acidity retention in date fruits (Figure 4).

### 3.6 Positive Correlations of Coatings and Packaging

**FIGURE 5:**
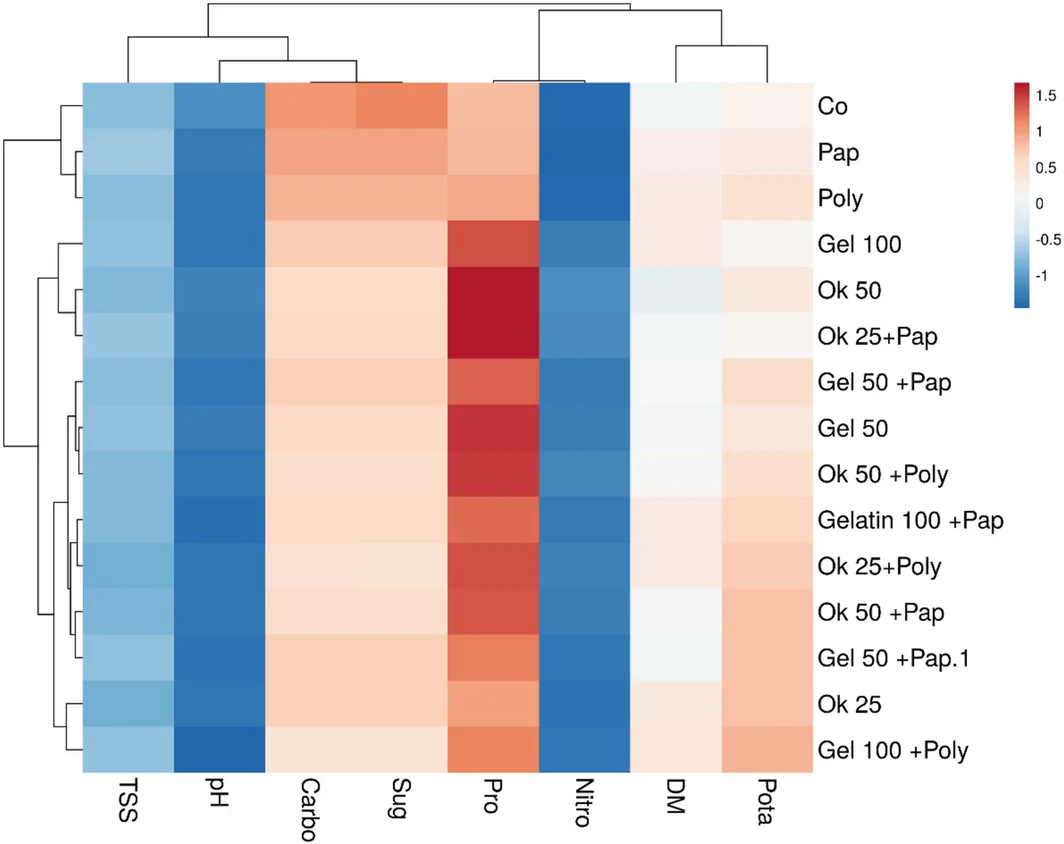
Effect of packaging type (paper bags, polyethylene bags, and uncovered control) and coating treatment (control, okra extract, gelatin coating) on postharvest quality parameters of Barhi date palm ( *Phoenix dactylifera* L.) fruits: Hierarchical clustering analysis (HCA) of Pearson's correlation coefficient (*r*) values of variable traits, where the color scale that indicates *r* coefficient values (*r* = +1 to −1) indicates positive (red) and negative (blue) correlations. Parameters include: PH; TSS; Sug: Sugar; Carbo; Carbohydrate; Pro: Protein; DM: Dry matter; Nitro: Nitrogen; Pho: Phosphorus, Gel: Gelatin; Poly: Polyethylene bags; Pap: Paper bags; Ok: Okra.

The protein content in date fruits demonstrated significant positive correlations to various treatments of interest; Okra 50 g L^−1^, Okra 25 g L^−1^ with paper bag, gelatin 50 g L^−1^, Okra 50 g L^−1^ with polyethylene bag, Okra 25 g L^−1^ with polyethylene bag, and Okra 50 g L^−1^ with paper bag. The correlation indicates effective protein preservation by okra extract and gelatin coatings with appropriate packaging material (Figure 5). The same can be said for phosphorus content, which also exhibited a strong positive correlation to gelatin 100 g L^−1^ with polyethylene bags, indicating it was the best treatment for phosphorus preservation, by a trait of significance (Figure 5).

## 4 Conclusion

The present study demonstrated that edible coatings based on gelatin or okra extract, particularly when combined with polyethylene packaging, were effective in preserving several physicochemical and nutritional attributes of Barhi dates during frozen storage. Among the tested treatments, gelatin at 100 g L^−1^ combined with polyethylene packaging showed the greatest overall effectiveness in maintaining fruit quality. However, further studies including sensory evaluation, microbial quality, and commercial storage conditions are required before practical recommendations can be made. These findings indicate that the combined application of edible coatings and packaging effectively preserved several quality attributes during frozen storage.

## Author Contributions

**Khalid. N. Asal:** conceptualization, investigation, project administration, supervision. **Zayad Amer Mustafaa:** data curation, project administration, methodology, investigation, formal analysis. **Heidar Meftahizade:** conceptualization, writing – original draft, formal analysis. **Kamil Atya Abed Sanjari:** conceptualization, resources, supervision, project administration, methodology. **Kh. A. S. Al‐Hamdani:** conceptualization, investigation, project administration, formal analysis, validation, methodology. **Raghad Adnan Ali AL‐Qady:** investigation, methodology, visualization, software.

## Funding

The authors have nothing to report.

## Ethics Statement

The authors have nothing to report.

## Consent

The authors have nothing to report.

## Conflicts of Interest

The authors declare no conflicts of interest.

## Supporting information


**Table S1:** ANOVA Table.

## Data Availability

Data available on request from the authors.

## References

[fsn372306-bib-0001] Abd Elwahab, S. M. , A. M. Abdallatif , A. E.‐R. A. Abd El‐Hafeez , and S. M. Soliman . 2025. “Efficacy of Alginate‐Silver Nanoparticle Coatings for Extending the Storage Period and Maintaining the Physico‐Chemical Parameters of Cold‐Stored ‘Barhi'Date Fruits.” Applied Fruit Science 67, no. 4: 1–11.

[fsn372306-bib-0002] Agostini, J. d. S. , S. d. P. Q. Scalon , K. E. d. Silva , F. F. d. Lima , A. P. E. Gomes , and M. M. Leite . 2013. “Physicochemical and Microbiological Characteristics of Minimally processed'Champagne'oranges ( *Citrus reticulata* × *Citrus sinensis* ) in Different Packgings.” Food Science and Technology 33: 84–92.

[fsn372306-bib-0003] Ahmed, A. R. , S. M. Aleid , and M. Mohammed . 2023. “Impact of Modified Atmosphere Packaging Conditions on Quality of Dates: Experimental Study and Predictive Analysis Using Artificial Neural Networks.” Food 12, no. 20: 3811. 10.3390/foods12203811 PMC10606818 37893704

[fsn372306-bib-0004] Al‐Habsi, N. 2025. “Date Palm ( *Phoenix dactylifera* L.) Fruit: Strategic Crop for Food Security, Nutritional Benefits, Postharvest Quality, and Valorization Into Emerging Functional Products.” Sustainability 17, no. 16: 7491.

[fsn372306-bib-0005] Ali, M. , A. Ali , S. Ali , et al. 2025. “Global Insights and Advances in Edible Coatings or Films Toward Quality Maintenance and Reduced Postharvest Losses of Fruit and Vegetables: An Updated Review.” Comprehensive Reviews in Food Science and Food Safety 24, no. 1: e70103. 10.1111/1541-4337.70103 PMC11734098 39812151

[fsn372306-bib-0006] Alqahtani, N. K. , S. A. Ali , and T. M. Alnemr . 2025. “Quality Preservation of Date Palm ( *Phoenix dactylifera* L.) Fruits at the Khalal Stage: A Review on Current Challenges, Preservation Methods, and Future Trends.” Frontiers in Sustainable Food Systems 9: 1558985.

[fsn372306-bib-0007] AOAC . 1990. “Official Methods of Analysis.” Journal of the Association of Official Analytical Chemists 62: 2742–2744.

[fsn372306-bib-0008] Chonhenchob, V. , K. Saha , S. P. Singh , and M. Siddiq . 2013. “Packaging Technologies for Dates and Date Products.” In Dates: Postharvest Science, Processing Technology and Health Benefits, 137–156. Wiley‐Blackwell.

[fsn372306-bib-0009] Dantas, T. L. , F. C. Alonso Buriti , and E. R. Florentino . 2021. “Okra ( *Abelmoschus esculentus* L.) as a Potential Functional Food Source of Mucilage and Bioactive Compounds With Technological Applications and Health Benefits.” Plants 10, no. 8: 1683. 34451728 10.3390/plants10081683 PMC8399980

[fsn372306-bib-0010] El‐Gioushy, S. , and M. Baiea . 2020. “Impact of Gelatin, Lemongrass Oil and Peppermint Oil on Storability and Fruit Quality of Samany Date Palm Under Cold Storage.” Bulletin of the National Research Centre 44, no. 1: 14.

[fsn372306-bib-0011] Felicia, W. X. L. , K. Rovina , M. N. Nur'Aqilah , et al. 2022. “Recent Advancements of Polysaccharides to Enhance Quality and Delay Ripening of Fresh Produce: A Review.” Polymers 14, no. 7: 1341. 35406215 10.3390/polym14071341 PMC9003407

[fsn372306-bib-0012] Frassinetti, S. , A. Castagna , M. Santin , et al. 2021. “Gelatin‐Based Coating Enriched With Blueberry Juice Preserves the Nutraceutical Quality and Reduces the Microbial Contamination of Tomato Fruit.” Natural Product Research 35, no. 24: 6088–6092. 32940058 10.1080/14786419.2020.1824224

[fsn372306-bib-0013] Gol, N. B. , and T. R. Rao . 2014. “Influence of Zein and Gelatin Coatings on the Postharvest Quality and Shelf Life Extension of Mango ( *Mangifera indica* L.).” Fruits 69, no. 2: 101–115.

[fsn372306-bib-0014] Goyal, K. , N. Singh , S. Jindal , R. Kaur , A. Goyal , and R. Awasthi . 2022. “Kjeldahl Method.” Advanced Techniques of Analytical Chemistry 1, no. 1: 105.

[fsn372306-bib-0015] Hasdar, M. , S. Nalinanon , N. P. Nirmal , T. Petcharat , and C. Sriket . 2026. “Sustainable Goat Skin Gelatin‐Based Edible Coatings Incorporated With Konjac Glucomannan: Physicochemical Properties and Preservation Efficacy on Strawberries.” Indonesian Journal of Science and Technology 11, no. 1: 59–80.

[fsn372306-bib-0016] Kamol, S. , J. Howlader , G. S. Dhar , and M. Aklimuzzaman . 2014. “Effect of Different Stages of Maturity and Postharvest Treatments on Quality and Storability of Pineapple.” Journal of the Bangladesh Agricultural University 12, no. 2: 251–260.

[fsn372306-bib-0017] Krueger, R. R. 2021. “Date Palm ( *Phoenix dactylifera* L.) Biology and Utilization.” In The Date Palm Genome, Vol. 1: Phylogeny, Biodiversity and Mapping, 3–28. Springer.

[fsn372306-bib-0018] Kumar, S. , and K. S. Thakur . 2020. “Active Packaging Technology to Retain Storage Quality of Pear cv.“Bartlett” During Shelf‐Life Periods Under Ambient Holding After Periodic Cold Storage.” Packaging Technology and Science 33, no. 7: 239–254.

[fsn372306-bib-0019] Mahara, G. , and A. Karki . 2024. “Packaging Materials and Their Effect on Shelf Life and Quality of Banana in Kailali District of Nepal.” South Asian Res J Agri Fish 6, no. 3: 42–52.

[fsn372306-bib-0020] Melton, J. R. , W. L. Hoover , P. A. Howard , and J. L. Ayers . 1970. “Atomic Absorption Spectrophotometric Determination of Boron in Plants.” Journal of the Association of Official Analytical Chemists 53, no. 4: 682–685.

[fsn372306-bib-0021] Metsalu, T. , and J. Vilo . 2015. “ClustVis: A Web Tool for Visualizing Clustering of Multivariate Data Using Principal Component Analysis and Heatmap.” Nucleic Acids Research 43, no. W1: W566–W570. 25969447 10.1093/nar/gkv468 PMC4489295

[fsn372306-bib-0038] Obón, C. , D. Rivera , A. Amorós , G. Díaz , F. Alcaraz , and D. V. Johnson . 2023. “Botany and Physiology of Date Palm.” In Date Palm: Production, Processing, and Nutritional Quality, 22–64. Wiley‐Blackwell.

[fsn372306-bib-0023] Olawuyi, I. F. , and W. Y. Lee . 2022. “Development and Characterization of Biocomposite Films Based on Polysaccharides Derived From Okra Plant Waste for Food Packaging Application.” Polymers 14, no. 22: 4884. 36433011 10.3390/polym14224884 PMC9692357

[fsn372306-bib-0024] Pillai, A. R. , A. S. Eapen , W. Zhang , and S. Roy . 2024. “Polysaccharide‐Based Edible Biopolymer‐Based Coatings for Fruit Preservation: A Review.” Food 13, no. 10: 1529. 10.3390/foods13101529 PMC11121366 38790829

[fsn372306-bib-0025] Saleem, A. , R. Rehman , S. Hussain , et al. 2023. “Biodegradable and Hemocompatible Alginate/Okra Hydrogel Films With Promising Stability and Biological Attributes.” International Journal of Biological Macromolecules 245: 125532. 37355067 10.1016/j.ijbiomac.2023.125532

[fsn372306-bib-0026] Santos Neto, J. P. d. , G. W. P. Leite , G. d. S. Oliveira , et al. 2018. “Cold Storage of ‘Palmer'mangoes Sorted Based on Dry Matter Content Using Portable Near Infrared (VIS‐NIR) Spectrometer.” Journal of Food Processing and Preservation 42, no. 6: e13644.

[fsn372306-bib-0027] Schanderl, S. 1970. Methods in Food Analysis, 701. Academic Press.

[fsn372306-bib-0028] Selim, M. , K. Nagy , and H. Mo . 2020. “Influence of Packaging and Cold Storage Conditions on the Physio‐Chemical Properties of Barhi Date Fruits.” Assiut Journal of Agricultural Sciences 51, no. 1: 79–91.

[fsn372306-bib-0029] Serra, S. , A. Goke , C. Diako , B. Vixie , C. Ross , and S. Musacchi . 2019. “Consumer Perception of d'Anjou Pear Classified by Dry Matter at Harvest Using Near‐Infrared Spectroscopy.” International Journal of Food Science and Technology 54, no. 6: 2256–2265.

[fsn372306-bib-0030] Sibomana, C. I. , A. M. Opiyo , and J. N. Aguyoh . 2015. “Influence of Soil Moisture Levels and Packaging on Postharvest Qualities of Tomato ( *Solanum lycopersicum* ).” African Journal of Agricultural Research 10, no. 12: 1392–1400.

[fsn372306-bib-0031] Tsegaye, K. Z. B. 2020. “Effect of Different Packaging Material on Shelf Life and Quality of Banana (Musa spp).” International Journal of African and Asian Studies 61, no. 1: 1–6.

[fsn372306-bib-0032] Uwiringiyimana, T. , S. Habimana , M. Umuhozariho , et al. 2024. “Review on Okra ( *Abelmoschus esculentus* (L.) Moench) Production, Nutrition and Health Benefits.” Rwanda Journal of Agricultural Sciences 3, no. 1: 71–87.

[fsn372306-bib-0033] Wang, Z. , Q. Zhang , D. Bukvicki , et al. 2024. “Konjac Glucomannan/Microcapsule of Thymol Edible Coating Reduces Okra Pericarp Browning by Regulating Antioxidant Activity and ROS Synthesis.” International Journal of Biological Macromolecules 276: 133641. 38969046 10.1016/j.ijbiomac.2024.133641

[fsn372306-bib-0034] Wieczorek, D. , B. Żyszka‐Haberecht , A. Kafka , and J. Lipok . 2022. “Determination of Phosphorus Compounds in Plant Tissues: From Colourimetry to Advanced Instrumental Analytical Chemistry.” Plant Methods 18, no. 1: 22. 35184722 10.1186/s13007-022-00854-6 PMC8859883

[fsn372306-bib-0035] Zhang, Y.‐L. , Q.‐L. Cui , Y. Wang , et al. 2021. “Effect of Carboxymethyl Chitosan‐Gelatin‐Based Edible Coatings on the Quality and Antioxidant Properties of Sweet Cherry During Postharvest Storage.” Scientia Horticulturae 289: 110462.

[fsn372306-bib-0036] Zhang, Y.‐L. , Q.‐L. Cui , Y. Wang , et al. 2021. “Effect of Edible Carboxymethyl Chitosan‐Gelatin Based Coating on the Quality and Nutritional Properties of Different Sweet Cherry Cultivars During Postharvest Storage.” Coatings 11, no. 4: 396.

[fsn372306-bib-0037] Zhu, X.‐m. , R. Xu , H. Wang , J.‐y. Chen , and Z.‐c. Tu . 2020. “Structural Properties, Bioactivities, and Applications of Polysaccharides From Okra [ *Abelmoschus esculentus* (L.) Moench]: A Review.” Journal of Agricultural and Food Chemistry 68, no. 48: 14091–14103. 10.1021/acs.jafc.0c04475 33205968

