# Supplementary material for: Enhancing Frozen Storage Stability and Nutritional Quality of Barhi Dates Using Okra Extract and Gelatin‐Based Edible Coatings Combined With Different Packaging Systems
Source: Food Sci Nutr. 2026 Aug 31;14(9):e72306. doi: 10.1002/fsn3.72306 (PMC13530378; doi:10.1002/fsn3.72306)
Supplement: Supplementary file 1 [file FSN3-14-e72306-s001.docx]

| Table S1. ANOVA Table | | | | | | | | | |
| --- | --- | --- | --- | --- | --- | --- | --- | --- | --- |
| Phosphorus | **Nitrogen** | **Sugar** | **Protein** | **Carbohydrate** | **Dry matter** | **TSS** | **pH** | **df** | **Source of variation** |
|  |  |  |  |  |  |  |  | 4 | Treatments |
| 0.0091 | 0.200 | 15.62 | 4.24 | 8.97 | 169.54 | 2.35 | 0.0539 |  | SS |
| 0.00228 | 0.0501 | 3.90 | 1.06 | 2.24 | 42.38 | 0.589 | 0.0135 |  | MS |
| 4.88 | 5.03 | 1.84 | 1.85 | 0.75 | 2.93 | 259.65 | 13.50 |  | F-calc |
| 0.0197 | 0.0181 | 0.199 | 0.197 | 0.579 | 0.0786 | 0.0001˂ | 0.0004 |  | p-value |
| * | * | *** | *** | *** | *** | *** | *** |  | Sig. |
| 0.0047 | 0.0997 | 21.26 | 5.74 | 29.94 | 144.80 | 0.0227 | 0.100 | 10 | Error. |
| 4.45 | 9.62 | 2.73 | 10.44 | 3.29 | 9.41 | 0.84 | 0.56 |  | CV (%) |
